# Law's Dilemma: Validating Complementary and Alternative Medicine and the Clash of Evidential Paradigms

**DOI:** 10.1155/2011/389518

**Published:** 2010-09-21

**Authors:** Ireh Iyioha

**Affiliations:** Faculty of Law, The University of British Columbia, Vancouver, BC, Canada V6T 1Z4

## Abstract

This paper examines the (in)compatibility between the diagnostic and therapeutic theories of complementary and alternative medicine (CAM) and a science-based regulatory framework. Specifically, the paper investigates the nexus between statutory legitimacy and scientific validation of health systems, with an examination of its impact on the development of complementary and alternative therapies. The paper evaluates competing theories for validating CAM ranging from the RCT methodology to anthropological perspectives and contends that while the RCT method might be beneficial in the regulation of many CAM therapies, yet dogmatic adherence to this paradigm as the exclusive method for legitimizing CAM will be adverse to the independent development of many CAM therapies whose philosophies and mechanisms of action are not scientifically interpretable. Drawing on history and research evidence to support this argument, the paper sues for a regulatory model that is accommodative of different evidential paradigms in support of a pluralistic healthcare system that balances the imperative of quality assurance with the need to ensure access.

## 1. Evidence-Based Medicine and Healthcare Regulation

States increasingly exercise their power to regulate affairs under their territories and determine the thresholds of societal expectations in accordance with the best available evidence. In the healthcare domain, there is increasing focus on evidence-based healthcare delivery, which involves the utilization of the presumed rationality of science [1] in healthcare management and professional regulation [2]. State law's espousal of science and the “circulation of meaning between science and law” have been denoted as central features of modernity [1]. Commentators suggest that the state has legitimated decisions based on scientific judgments; in these cases, “scientific judgments glide into normative judgments” [1, 3]. There is little wonder that many policy decisions are increasingly being founded on scientific evidence, especially when the decision to be made concerns clinical practice guidelines that focus on specific individual treatments [2]. In the legitimization of *new* or pre-existing health systems, this new standard narrative has not been different. In the regulation of the paradigm known in Western communities as “Complementary and Alternative Medicine”, state law must decide between competing versions of reality—the scientific (generated by a biomedically-based Randomized Clinical Trial) and the clinical (generated through diverse nonbiomedical methods) and reported by ardent health consumers. 

**Figure 1:**
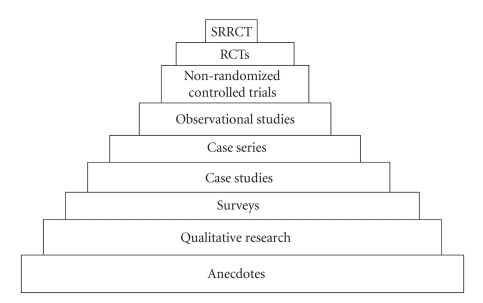
Hierarchy of scientific evidence.

Complementary and Alternative Medicine (“CAM”) is an inconvenient reality in today's medical practice. Its gradual re-emergence and prevalence in biomedical and scholarly discourse has generated controversies regarding the issues of safety and cost effectiveness, and the basis for which the state should grant legitimacy to CAM. A recurrent inquiry is whether CAM can be scientifically validated. This implies that CAM must be amenable to scientific patterns of evidence. Ironically, the lack of evidence of safety and efficacy for many CAM therapies has had little impact on consumer patronage. However, in the discourse on state regulation of CAM, some commentators argue that regulation must be based on scientific evidence of safety and efficacy. This inquiry is not surprising since healthcare policymakers are increasingly interested in “developing and implementing evidence-based decision making” [2]. Evidence-based Medicine (“EBM”) has been defined as the “conscientious, explicit, and judicious use of current best evidence in making decisions about the care of individual patients” [4]. It also denotes the integration of “individual clinical expertise with the best available external clinical evidence from systematic research” [4]. Within the EBM paradigm, the Systematic Reviews of Randomized Controlled Trials (“SRRCTs”) sits at the highest level of the hierarchy of evidence for establishing the safety and efficacy of healthcare modalities [2].  Figure 1 below illustrates the other evidential paradigms in descending order.

The importance of approval through the SSRCTs is manifest in its influence in judicial, economic, and policy-making circles. For health professions to obtain recognition by the dominant political, economic, and judicial structures, they must demonstrate commitment to the public welfare and be associated with or supported by science [5]. According to Casey and Picherack, some conventional health professional organizations and commentators have taken the position that CAM practitioners should not be recognized by the state in the form of self-regulation unless and until they can demonstrate that their therapies or practice methods are evidence based [6]. Thus, the concept of evidence-based medicine looms largely at the centre of discrediting CAM, and it is, therefore, fundamental in the barriers to the recognition of this form of medicine [6].

## 2. The RCT and CAM

The RCT is specially designed to validate categories of medical interventions that are observable and measurable. There are dissenting opinions on the role of the RCT in determining what interventions are efficacious. Some arguments have highlighted the structural incompatibility between the RCT, which was designed for biomedical research, and CAM, which primarily comprises therapeutic philosophies that transcend the biomedical. Other arguments have simply drawn attention to problems inherent in the RCT methodology itself, which render it a less than perfect methodology for researching many therapies, whether biomedical or CAM. In the former case, the argument is that the very nature of CAM disqualifies the RCT as a suitable research methodology. 

CAM constitutes a broad range of holistic and integrated etiologies of illness and healing that incorporate “complex causal networks” and unconventional concepts such as “bioenergetic homeostatis, repressed memories, and spiritual disturbances” amongst others [7]. The practice of CAM is based on “complex” and “personalized” [7] episodes between practitioner and client, in which the treatment regime is specifically tailored to suit the particular interests of the client. These regimes can differ considerably from one client to another, “both in the substance of their contents and in their methods of application” [7]. This may lead to varying individual responses, “possibly even for the same person,” though at different treatment episodes [7]. Furthermore, in chronic disease and prevention cases, CAM is usually administered over reasonably long periods [7], and this may not fit with the methods of conventional research. 

Against this background, this paper evaluates competing theories for the regulation of CAM—science-based (statutory) regulation on the one hand, and multilayered investigative methods, which incorporate the best of the RCT with perspectives from other fields such as anthropology, on the other. The paper specifically discusses anthropological research methods for the assessment of CAM, which highlight the philosophical side of CAM rather than the pathophysiological. Adopting ethnography as an evidential tool, anthropological research in CAM emphasizes qualitative data collection techniques, focus group strategies, and observation of patient-practitioner interactions. Notably, the objective here is not to discard the utility of scientific regulation, but to highlight its limits and support comprehensive proposals through which the law can generate the needed evidence to undergird the regulation of different CAM therapies, particularly those that are not easily amenable to the RCT methodology. 

As part of the discussion of the RCT methodology for validating CAM, the paper summarily examines the historical evidence on the impact of a science-based regulatory framework on the development of some statutorily regulated CAM modalities, such as osteopathy and chiropractic. A close examination of the mechanism of the RCT reveals that the statutory or medical model of regulation, which fundamentally espouses science as the objective mode for validating medical practice, will have a significant impact on the development and practice of CAM. The statutory model necessarily involves the institutionalization of medical practice standards, which do not often correlate with those operable in the practice of CAM. In fact, a temporal examination of legal regulation reveals that CAM interventions whose underlying mechanisms have a high probability of efficacy and are amenable to scientific enquiry have achieved state recognition. 

However, this recognition has often come with a price tag. The statutory recognition of osteopathy and chiropractic in Britain mandated the rejection of the esoteric foundations of these therapies. It also mandated the exclusion of the nonspecific elements and philosophies that accompany the practice of these therapeutic systems, which had hitherto formed the basis of their popular acceptance in the originating communities. The RCT's limitations in investigating nonbiomedical and subjective experiences can lead to the restructuring of nonbiomedical healthcare systems, which would have evolved to be unique and well-defined systems that are intrinsically different from biomedicine. The effect of an application of a methodology designed for a specific kind of research on another entirely different set of medical enquiry can be the loss of key aspects of the latter system. Indeed, the historical evidence as examined in this paper shows that in many cases, the CAM therapy becomes a substrate of biomedicine.

Based on this evidence, this paper contends that while scientific validation is laudable and beneficial, yet an uncritical application of the RCT methodology to *all* CAM interventions will be less than an optimal framework for regulating CAM. The contentions in this paper are primarily grounded on the dual impacts of the proscience evidence-based regime on the development of CAM, as well as on anthropological evidence and the histories of osteopathy, acupuncture, homeopathy, and chiropractic in the UK. The paper argues that given its limitations, dogmatic adherence to the RCT as the exclusive methodology for legitimizing CAM will have multiple impacts on the status of CAM. While one of these will predictably be a beneficial impact in its emphasis on outcomes rather than explanations [8], an outcome that will elevate CAM to the status of an accepted healthcare option, this paper indicates that this same benefit interpreted in a historical context will constitute a drawback for the development of some CAM modalities. Beyond imposing a narrower scope of practice on CAM practitioners and mandating the abandonment of several distinctive notions that distinguish CAM from biomedicine, the use of science as an exclusionary tool for therapies that are not amenable to the RCT might result in the gradual effacing of CAM and its equation with biomedicine. This will hardly be in the best interest of consumers, especially those who are attracted to CAM because of its holistic and patient-centred approach to healthcare.

Therefore, the paper sues for a regulatory system that is accommodative of different evidential paradigms. It suggests that the acceptable evidence must be that which takes into account the unique nature of CAM and advocates for a modified methodological framework, which acknowledges the belief systems and values inherent in CAM as part of the therapeutic process itself. However, it is noteworthy that the arguments proffered in this paper do not neglect the fact that studies have shown that about 30%–40% of physician recommended services lack evidence of effectiveness or have very little compelling evidence to support the therapeutic claims [9]. While acknowledging that many biomedical services and products are yet to meet the threshold of the EBM paradigm, this paper focuses on the impact of the EBM paradigm on CAM because the medical and scholarly communities continue to endorse this paradigm, through the RCT, as the optimal approach to validating CAM.

The analysis begins in Section 1 with a brief discussion of the placebo effect. In Section 2, the scientific evidential paradigm is discussed with particular focus on the role of the RCT in evaluating CAM practice. An evaluation of the RCT necessarily implicates the Bayesian theory, a theory within medical epidemiology and philosophy, which captures how existing evidence interacts with (and may influence) emerging evidence to produce new outcomes. This theory is examined to reveal its centrality to statutory regulation of healthcare professions. Historical examples are drawn from the early prohibition and regulation of osteopathy, chiropractic, homeopathy, and acupuncture to establish the nexus between the Bayesian theory and statutory regulation of healthcare modalities.  Section 3 examines anthropological research methods for validating CAM, focusing on the arguments of medical anthropologists who question the objectivity of scientific forms of evidence through differently constructed forms of evidence [10]. The concluding part of the paper addresses the need for a validation process that recognizes the inherent duality of approaches, methods, and belief patterns between CAM and biomedicine.

## 3. CAM and the Placebo

The increase in CAM patronage and of constitutional claims to tax relief and reimbursement for CAM expenses has influenced debates about cost effectiveness, an index that has emerged as a crucial determinant of which CAM modality should obtain state funding. In the debate on the cost effectiveness of CAM, commentators have called upon CAM practitioners to provide evidence of the superiority of their therapies over the placebo [11]. The term “placebo” pervades most discussions of the therapeutic effects of CAM. In fact, the subject of CAM appears inseparable from that of the “Placebo Effect” [12]. Although there is substantial literature on the precise medical definition of the term [13, 14], yet there is significant disagreement among physicians, clinicians, psychiatrists, and philosophers of science on what constitutes a placebo effect [15]. It is usually agreed to be a nonspecific substance, with no specific curative effect, given to satisfy the psychophysiological needs of a patient. As a substance of operant conditioning, the placebo is also “used on a control group in experimental design to further test the efficacy of an active substance or drug” [13, 14]. Placebo Effect (“PE”) has been defined as “the bodily change due to symbolic effect of a treatment or treatment situation and not its pharmacologic or physiologic properties” [12]. This definition accommodates a PE achieved by other causes outside the pharmacologic properties of the drug. Such a broad definition of the PE creates room for some CAM modalities such as mind-body healing and meditation [12].

While exponents of CAM may raise the question of scientific efficacy as a matter of genuine scientific curiosity [12], some commentators devoted to this enquiry have argued that CAM should not be exempt from rigorous testing and should be examined using the same methods used to test conventional therapies [16]. Brody, in a discussion of the placebo effect in the study and practice of CAM, asserts that the chasm between CAM and biomedicine—insofar as scientific efficacy goes—may be much narrower than advocates of biomedicine would like to believe [12]. Indeed, some scholars contend that some complementary therapies are so close to orthodox medicine that they can conveniently be regulated along the same frameworks [17]. However, given the esoteric nature of some CAM interventions, it has been argued that the therapeutic approach of holistic therapies must take into account the possibility of emotional and psychological harm, which must be considered in the choice of a regulatory scheme [17]. While notions of harm have been heavily influenced by legal and biomedical rules [17], legal regulation of CAM faces hurdles because the legal and biomedical professions, much like the RCT methodology, lack guidelines to exercise hegemony over notions of harm “beyond the quantifiable, measurable notion of harm” [17]. 

It is noteworthy that CAM practice is common in degenerative medical conditions and those where behavioural, emotional, or spiritual factors play a major role; in these areas, the introduction of scientific logic into medicine has not produced “noticeable improvements and (have) in fact led to deterioration” [18]. Thus, in considering the RCT as a research tool, the amenability of CAM therapies (especially those that profess to heal the emotional facet of disease) to the RCT needs to be factored into the choice of a research methodology.

## 4. Evaluating the RCT

Federal regulatory agencies have the responsibility to determine the safety and efficacy of medicines, nutritional supplements, and herbal medicines. This function is critical to the health and wellbeing of consumers who are unable to assess the safety and quality of health products. Indeed, the healthcare market is described as imperfect because consumers lack the requisite tools to assess the goods on sale since the exercise of their judgment is contingent on factors such as test results [19], quality assessment, and the professional judgment sometimes required before purchase. Thus, statutory regulation of CAM is not simply about granting professional recognition to CAM practitioners; it is first about protecting consumers' health and enabling them to make informed choices. A further objective of regulation is that CAM professionals will acquire the status necessary to obtain funding/research grants and collaborate with biomedical professionals to ensure the best healthcare that would meet population needs, especially in rural and underserved communities with a high rate of inequitable healthcare delivery.

The recognition conferred by statute raises a specific CAM modality to an accepted healthcare option [17]. The passing of the Osteopaths Act in 1993 and the Chiropractors Act in 1994 in the United Kingdom has been interpreted as significant landmarks. While many CAM therapies are voluntarily self-regulated, the passing of similar Acts in other countries has generated a lot of optimism that many CAM therapies can achieve statutory recognition. However, the process leading to the grant of statutory legitimacy necessarily requires favourable evidence that the healthcare intervention is effective against the (range of) conditions for which it is directed [7]. In order to establish this evidence, the Randomized Clinical Trial, which is usually controlled and double blind, is applied to the given data [20].

The Randomized Controlled Trial is a quantitative study in which people are “allocated at random” to receive one of several clinical interventions [20]. One of these interventions is the standard of comparison or control, and it may be a standard therapy or practice, a placebo, or no intervention at all [21]. When evaluating the results of an RCT, the methodological quality of the design, the conduct of the trial, and the influence of pretrial beliefs are important points to be considered. Hrobjartsson and Brorson argue that these factors make it imperative to interpret RCT results cautiously [21]. Indeed, where it is likely that a trial has design inadequacies, or when the intervention is based on theories that are not scientifically interpretable, the results of the trial must be viewed with caution [21]. 

Before discussing the RCT in detail, it is important to identify limitations to the effectiveness of the RCT in measuring the efficacy of interventions in medical science generally. One opinion is that the RCT is not an unassailable methodology for testing CAM because the trial will have to confront the same problems inherent in testing orthodox interventions through RCTs [12]. Howard Brody argues that as long as the placebo effect remains a significant part of the results of biomedical trials, the RCT will be an inadequate method for discountenancing CAM [12]. Brody notes that since the RCT supposedly controls for all “nondrug factors”, it assumes that other possible causative factors are irrelevant to the medical scientist; as a result, the “nonscientific” elements of CAM—the belief and thoughts of the patient, the multifaceted etiologies of disease causation and treatment that produce a placebonic effect—are excluded from the trial [12]. 

An important point in Brody's argument is that the placebo effect remains present in RCTs of biomedical interventions even though the trial is designed to consider all nondrug factors as irrelevant. Beyond this, we must note the variable factors that could influence the result of a clinical trial. Results could be influenced by the effect of the interventions, inadequate methodological design, bias, or chance [21]. The conduct of a trial by data or outcome interpreters with a high interest in the results of the trial may be a source of bias [21]. While genuine intent may be an abstract term, it has been suggested that data or outcome interpreters will need to have such intent, untainted by strong feelings for a special result from the trial [21]. 

Logistical challenges in RCTs are another problem. Pharmacological standards require that a trial be conducted according to the standards of good clinical (research) practice, and this very often refers to a process of auditing [21]. Systematic reviews of homeopathy trials where the methodological design was studied have revealed defective logistical designs and a general low trial quality [21]. Hrobjartsson and Brorson have observed that because quality analyses of RCTs are often based on “analyses of trial reports and not the conduct of the trial”, key aspects of trial methodology or the problems involved may not be described. In one study of a broad group of trials, with binary, objective outcomes, it was observed that there was unconcealed allocation and no double blinding, such that the participants were able to predict the treatment they would receive before the start of the trial [22]. As Greschner has noted, “the “evidence” in evidence-based decision making can be collected or interpreted in unfair or biased ways; if the evidence is biased, so, too, will be the policy or guideline” [2]. 

Finally, Hrobjartsson and Brorson, like Brody, have noted that the RCT discounts the metaphysical in many CAM modalities. While these elements arguably have no effect beyond the placebonic, yet they are important to any study because the placebo itself has been described as medicinal [23]. Indeed, “the phenomenon labelled “placebo effect” in RCTs is recognised as a powerful intrinsic component of alternative healing” [10]. 

Toke Barfod's concept of fragility sums up the above discussion [24]. Barfod makes a distinction between CAM interventions that are akin to biomedicine and those whose belief systems differentiate CAM from conventional medicine. While the former includes CAM therapies like herbal medications that are considerably independent of the patient's belief system, the latter includes interventions that depend on the faith or belief system of the patient and practitioner. Barfod places CAM interventions along a spectrum with these two divisions of CAM lying at the two ends of the spectrum. These divisions can be classified as the “context/belief-independence” systems and the “context/belief-dependence” systems [12, 24], with the latter being fragile therapies, and the former, nonfragile [12, 24]. The argument is that the RCT is an effective way to validate therapies at the non-fragile end of the spectrum. However, as the therapy progresses towards the fragile end of the spectrum, the RCT fails to be an effective tool for assessment [12, 24]. The RCT creates an environment foreign to the cultural and philosophical belief context within which the fragile therapies would be most effective [12, 24]. This argument is akin to that proffered by anthropologists and ethnographers, who contend that the unique nature of CAM favours an evidential paradigm that is cognizant of the effect of philosophies, culture, and/or belief systems on the efficacy of the treatment [10]. 

A related issue to the above limitations of the RCT is that of pretrial belief. A closer evaluation of pre-trial belief in the RCT reveals its centrality to the results of a trial. In this paper, pre-trial belief is analyzed to highlight its nexus with statutory regulation of health systems. I employ the Bayesian Theory, a concept within medical epidemiology and philosophy, to illustrate this nexus. Apart from rationalizing the affiliation between scientific validation and statutory regulation, the Bayesian Theory is also foundational to advocating for diverse legal supervision of CAM beyond regulation based solely on results of trials that strictly adhere to the RCT model. However, before addressing the Bayesian Theory, it is pertinent to discuss the role of pre-trial belief (or the null hypothesis) in RCTs and the limitations inherent in its interpretation of the efficacy of a therapy.

## 5. Testing CAM: The Null Hypothesis and Its Limitations

The process of setting up and testing a hypothesis is fundamental to statistical inference [25]. In the process of setting up a test, a theory is put forward “either because it is believed to be true or because it is used as a basis for argument, but has not been proved” [25]. Thus, a null hypothesis “represents a theory that has been put forward as a basis of argument,” but that is yet to be proven [25]. An example is the claim that a CAM modality is better than the conventional therapy for the treatment of the same or similar symptoms. Special consideration is given to the null hypothesis in a clinical trial [25]. This is because “the null hypothesis relates to the statement being tested, whereas the alternative hypothesis relates to the statement to be accepted if or when the null is rejected [25].” Therefore, the result of the test is usually expressed in terms of the null hypothesis [25]. This implies that the null hypothesis could be either “rejected in favour of the alternative hypothesis”, or the conclusion may simply be “do not reject null hypothesis” [25]. This is because a conclusion that the null hypothesis should not be rejected does not mean that the null hypothesis is true; rather, the implication is that there is no sufficient evidence against the null hypothesis in favour of the alternative [25]. According to Easton and McColl, “rejecting the null hypothesis suggests that the alternative hypothesis may be true” [25].

There are a number of limitations in the theory of the null hypothesis. The most significant for this study is that an acceptance of the alternative hypothesis “only commits us to a difference in observed parameters; it does not prove that the theory or principles that predicted such a difference are true, since it is always possible that the difference could be due to additional factors not recognized by the theory” [26]. This point is significant for CAM therapies that combine multiple factors (many of which are not recognized by the theory underlining the trial) as part of the healing process. Put plainly, many CAM interventions are “complex and multistranded” and may require a diverse range of behavioural changes, such as life style or diet changes [10], which are complementary to the primary CAM therapy being administered to the patient. These complementary strategies are not usually factored into the trial process, and the impacts of such strategies are, therefore, not reflected in trial outcomes. 

An additional problem arises where there is strong belief in the alternative hypothesis at the outset of the trial. This makes the null hypothesis the reverse of what the experimenter actually believes; the implication is that the null is put forward only to allow the data to contradict it [26]. This is usually the case where the data interpreter has no genuine interest in achieving a positive result in the trial of a CAM therapy. Other concerns relating to the null hypothesis or pre-trial belief in the RCT are set out in the Bayesian theory. The next section examines these concerns.

## 6. The Bayesian Theory and Statutory Regulation

Within medical epidemiology and philosophy, evidence is evaluated within a continuum of already existing information. Therefore, the interpretation of new evidence depends, to some degree, on what is already known of the intervention [21]. The existing information constitutes old evidence, which interacts with the new to produce what becomes the clinical result. This phenomenon is termed the “Bayesian Perspective”. The framework of this theory is that “observers evaluate new evidence in the light of their background knowledge” [21]. In the process of evaluating new medical evidence, there exists an estimated probability that a given result will occur, and this is called the “prior probability” [21]. The prior probability “cannot be neglected when interpreting new evidence” [21]. At the other end of the continuum is the “posterior probability”. The posterior probability is evaluated on the basis of the prior probability and the strength of the new evidence. When prior probability is low, a significant amount of positive evidence is needed to change posterior probability [21]. Thus, pre-trial beliefs, coupled with the underlying theories in CAM, play a major role in randomized controlled trials. Given this situation, when the prior probability is based on an implausible mechanism or is scientifically illogical, the prior probability falls towards zero. Given the complex causal philosophies of many CAM interventions, the tendency has been for the prior probability of CAM to be estimated as very low, such that the posterior probability in CAM trials hardly changes [21]. Hrobjartsson and Brorson assert that this makes the RCT a nonneutral “algorithm” for ascertaining the effectiveness of a clinical intervention [21]. 

Furthermore, it is noteworthy that the focus in a clinical trial is on the existence or lack of therapeutic effect of the intervention and not the underlying mechanism [21]. In other words, the hypothesis is not about “how” it works, but “whether” it does. Willis and White have explained that in the RCT, it is the outcome of an intervention that is important and not the “underlying paradigm of disease causation or treatment” [8]. However, it is significant that in the interpretation of a clinical trial, there is a close relationship between the probability of the underlying mechanism of action and the projected prior probability of the intervention [21, 27]. This implies that when the postulated mechanism of action for an intervention is implausible or not scientifically interpretable, the prior probability of the intervention to have any observable clinical effect falls [21]. When the underlying mechanism is considered seriously defective, the prior probability of the intervention to have “significant” and observable clinical effects falls to zero [21]. If the prior probability for an intervention is zero, such as the case of homeopathy, a problem arises. According to Hrobjartsson and Brorson, in such a case the interpretation of evidence breaks down [21]. 

Interestingly, homeopathy is not statutorily regulated in many countries. The homeopathic theory of disease has been described as “absurd according to the standard scientific position” [21], such that the trend has been to ascribe a prior probability of zero to its hypothesis. The result is that the clinical trial results of homeopathy are impossible to read [21]. In contrast to homeopathy, while osteopathy, acupuncture, and chiropractic are also based on theories significantly foreign to biomedical science, their theories of disease have been accepted as postulations not entirely absurd to Western science [21]. As earlier noted, these CAM interventions have been granted statutory recognition in some countries, such as the United Kingdom and Australia. In the next subsections, I examine briefly the historical charts of these therapies, from preregulation to postregulation, and highlight the compromises the systems have had to make in order to obtain legal recognition.

### 6.1. Osteopathy and Chiropractic

A historical evaluation of statutory regulation of some CAM therapies reveals a process of reductionism from esotericism to medical exotericism. History tells of how medical antagonism to osteopathy intensified in the 1920s [28]. In 1931, the practitioners sought state recognition through registration, but the medical profession opposed on the basis of “the lack of empirical evidence for the existence of osteopathic lesions” [28] and the level of training of the practitioners. In 1935, a statement signed by 800 medical and biomedical scientists was submitted to a Select Committee of the House of Lords, denouncing the lack of scientific evidence for the theoretical underpinnings of osteopathy [28]. The British Medical Association laid emphasis on the incompatibility between osteopathic and “modern medical concepts of the nature of pathogens” [28].

However, with the approval of the medical profession, the Osteopaths Act was passed in Parliament in 1993. It provided for state registration but at a cost. The osteopathic profession had to abandon its claims and agree to be defined “as a generic form of medicine” [28]. Having streamlined its practice to a framework acceptable to the medical profession, it progressed from being a threat to becoming a system of healthcare that was supplemental to medical care in the area of musculo-skeletal problems [28]. On this note, Cant and Sharma have opined that “in Britain, the story was to be acceptance with subordination rather than acceptance with amalgamation” [28].

There are a number of similarities between the Osteopaths Act and the Chiropractors Act of 1994 in the United Kingdom. The details of both Acts are almost identical, and both are modelled after the Medical Act of 1983 [17]. The Chiropractors Act originated after a working party was set up to determine the feasibility of statutory recognition of chiropractic [17]. A significant reason for the promulgation of the Act was the positive result of a Medical Research Council investigation via randomized control trial into the use of chiropractic care for the treatment of lower back pain. The trial weighed the results of chiropractic against conventional treatment [17]. 

When we analyze the statutory recognition of osteopathy and chiropractic in the light of Barfod's notion of fragility, it is obvious that osteopathy and chiropractic are at the non-fragile end of the spectrum. Whatever “nonmedical” factors may have placed them at the other end of the continuum have been eliminated from their therapeutic theories so they can fit into a statutory regulatory framework.

### 6.2. Homeopathy and Acupuncture

Homeopathy came under attack from the medical profession in the second half of the nineteenth century [28]. It was argued that the homeopathic theory of disease treatment was absurd to medical theories. This led to attempts in the 1950s to have homeopathic practitioners charged in the law courts for the death of patients under their care [28]. Interestingly, with the rise in the attack on their theory of practice, homeopathic practitioners began to adopt more allopathic techniques. Nicholls aptly captures this process as the “bastardisation of homeopathy” [29]. According to Cant and Sharma, “homeopathy as practiced by the doctors trained in the Faculty of Homeopathy in London became a tolerated if insignificant and marginalized group within the broader medical profession, and when the National Health Service was established in 1948, homeopathy was grudgingly accorded a foothold within it” [28]. 

A similar process of metamorphosis can be seen in acupuncture, a generic therapy which originated in China. In the early nineteenth century, it was practiced by both medically and nonmedically qualified practitioners in Britain [28]. While it was practiced in its original form with its gamut of Chinese therapeutic/philosophical traditions in China, the practice of acupuncture to be acceptable in Britain had to distance itself from some of its theories. According to Cant and Sharma, “acupuncture as “naturalized” in Britain was divested of its classical theoretical underpinnings in Chinese diagnostics and understandings of the human person” [28]. From the mid-nineteenth to mid-twentieth century, medical journals showed little interest in acupuncture and denounced “nonmedically qualified acupuncturists” [28]. Fulder identifies the attempt by medical acupuncturists to interpret acupuncture's effect on pain in biomedical terms and theories of the nervous system [30].

It is important to emphasize that the process of scientific validation modified not only the theoretical underpinnings of the intervention, but also the practice and range of conditions to which it can be applied. Thus, “medical acupuncture has been compartmentalized to the extent that it is used for a restricted range of conditions” [28]. However, this “very compartmentalization has served to distance doctor acupuncturists from the growing body of nonmedically qualified acupuncturists who practice acupuncture as a more generic therapy and who regard themselves as more faithful to the original Chinese therapeutic tradition” [28]. An important point that arises here is that nonmedical practitioners continue to practice acupuncture, employing methods adversative to biomedically accepted methods with the knowledge that these methods, which are devoted to the Chinese therapeutic tradition, are the primary attraction for consumers. If consumers will continue to patronize CAM whether or not accepted by biomedical science, then the law must devise a way to protect them from whatever harm may be inherent in the interventions. The next section examines anthropological research methods, which investigates CAM through a different evidential paradigm.

## 7. Anthropological Evidence

Anthropological research methods differ considerably from the scientific. The focus of the research is neither predetermined nor tightly structured [10]. The methodology employs an observer situated in the context of the phenomenon under observation [10]. Anthropologists argue that ethnographic research allows discovery of important factors affecting the results of a research, which are not visible through scientific methods. Ethnographic research looks primarily into the interaction between a particular patient and their specific healthcare provider [10]. According to Christine Barry, ethnographic research utilizes “embodied” and “intersubjective data” [10]. Phenomena are analyzed over long periods and fieldwork stretches over long timescales [10]. The anthropologist's main tool for investigation is himself or herself. Therefore, the collection, evaluation, and interpretation of evidence are not through randomization, standardization, or blinding techniques, but through personal, intuitive patterns of knowledge. In an RCT conducted on homeopathy, Weatherly Jones reports that trial practitioners were of the view that the blinding “interfered with their normal practice routines, to produce a radically different version of their normal therapeutic practice” [31]. In homeopathic training, a remedy to be active has to match the total symptom picture to that of the patient. While there can be a shared diagnosis of an ailment between biomedicine and CAM, many biomedical prescriptions for the same ailment will be “useless” and “inert” for the patient in homeopathic theory [31].

Barry asserts that in the view of nonbiomedically trained CAM practitioners, the evidence needed to validate CAM is that which investigates not whether a therapy is working according to biomedical/scientific criteria, but whether it is making a difference to the bodies, beliefs, and social and cultural experiences of its clients and whether patients keep coming back [10]. Furthermore, she asserts that “anthropological and other forms of evidence may prove to be political tools to assist in this enterprise of transformation” [10]. The anthropological perspective to CAM validation affirms the shortcomings of the RCT already highlighted above. Anthropological forms of evidence also confirm the argument in this paper that policymakers need to employ a variety of evidential paradigms to regulate CAM. Specifically, policymakers need to factor consumers' values into the decision-making process. This is particularly important when we consider the benefits that consumers claim to derive from using CAM. The anecdotes of efficacy often form the core reason for litigations over reimbursement for CAM use. 

However, it is important to note the limitations of anthropological research methods. One of these limitations is that it de-emphasizes the significance of scientific validation in “non-fragile” CAM interventions, such as in the case of herbal medicines, which share a closer quality with conventional medicines [10]. Clearly, ethnography is not relevant for all CAM interventions. Therefore, a superimposition of this evidential paradigm above all others will fall to the same fallacy associated with the claim that the RCT is the best method for validating a medical intervention.

Finally, it is pertinent to highlight Willis and White's sociological analysis of the implications of the EBM theory for the future development of CAM. According to the authors, EBM will mandate the use of the RCT in validating CAM, which itself will be ineffective for CAM interventions lacking an acceptable evidential base [8]. Furthermore, CAM therapies found to be effective through the RCT may be incorporated into biomedical practice, thereby losing their “alternativeness” to biomedicine. Citing acupuncture and chiropractic in the Australian context as examples, the authors argue that there was serious attempt to restrict the practice of these therapies to orthodox practitioners [8]. 

However, Willis and White conclude that EBM will not play a decisive role in CAM regulation because the history of health services reveals that “as a basis for politico-legal legitimacy” clinical legitimacy (i.e., the continuous patronage of a therapy by consumers willing to pay for it) is more significant than scientific legitimacy [8]. Using chiropractic as an example, they assert that the reason for the survival of the modality is not due to scientifically acceptable evidence; rather, it is due to clinical legitimacy—the belief of patients who experienced relief from it. Another example may be found in the clinical efficacy of psychoanalysis. The acceptance of psychoanalysis has been based largely on its clinical efficacy irrespective of the controversies regarding the authenticity of its theories. The authors conclude that “social processes external to the health system” would constitute the primary push factor that would influence the state to regulate a given therapy [8]. Drawing examples from the registration scheme for Traditional Chinese Medicine (“TCM”) and natural therapies in Australia, they highlight the lack of connection between the patchy results of RCTs conducted on TCM and the passing of an Act to regulate TCM. 

This analysis is significant here in its identification of the influence of patient choice in health systems regulation. The understanding is that there will be consumers whose decisions to patronize a particular CAM modality may not be affected by the fact that the modality is unregulated. This point is strengthened by the fact that CAM has been interpreted as a manifestation of changes in medicine's institutional authority initiated by a consumer-driven healthcare environment [32] and by the (re)new(ed) patient interest in having their values incorporated into healthcare decision making, and healthcare delivery. If, as has been noted, consumer protection is imperative in an imperfect healthcare market, then it implies that states must devise a more inclusive validation method for CAM that transcends the limits of the RCT.

## 8. Conclusion: The Need for a Pluralistic Evidential Paradigm

The foregoing discussion has focused on highlighting the limitations and strengths of different evidential paradigms for regulating CAM. It has been noted that the RCT, while beneficial in its approach to medical research, is not itself designed to provide optimal outcomes in researches that combine subjective feelings and experiences with the pure medical effect of a therapy. As some scholars have noted, “science is structured to remove any human factors from the context of the study, setting up a model that is detached from feelings, meaning and subjective experiences” [33]. Since research methodologies “are not considered to be independent from their paradigm of reference”, it can be said, therefore, that “the methods used…for conventional medical research reflect the paradigm on which they were founded” [7]. 

Other scholars have noted that the adoption of a single evidential paradigm for CAM is less than optimal. Lewith et al. assert that “no single research methodology in itself yields all the knowledge necessary with respect to effectiveness, efficacy, safety, and patient/doctor treatment preferences” [34]. Vickers affirms that the RCT indeed “does not aim to” provide the answer to “all questions of interest in health care” [35]. These views have prompted some scholars to demand for multiple research methodologies. Wayne B. Jonas voices the following concern.

Is it possible to develop a pluralistic approach to research methods that retains the value of Western science for medicine and yet respects the diversity of radically different concepts about life, health, and service [36]?

This paper contends that such an approach is possible and should be pursued. The evidence collated in the foregoing discussion favours a research paradigm that recognizes the structural limits of the RCT in the context of evaluating nonbiomedical therapeutic systems. This approach takes the very weaknesses of the RCT as a starting point for the investigation of CAM. This paper advances such a modified framework, which acknowledges and integrates the belief systems and values inherent in CAM as part of the therapeutic process itself. These beliefs and values have historically been denigrated as “placebo”, a term which implicitly reaffirms popular conceptions about CAM within Western biomedicine. A randomized clinical process that is (re)designed to integrate the CAM paradigm, a paradigm that seeks its therapeutic strengths from within and without the medical effects of the therapy itself, ensures the transfer of knowledge between medical systems. 

As reiterated in the foregoing discussion, science sits at the root of the efficacy, safety, and regulation debate on CAM. Historically, state law has been known to espouse the dictates of science, and science, bolstered by the force of law, has been deployed as a tool of exclusion of nonwestern medical norms. With the increasing utilization of CAM by a significant proportion of society, it is time for states to embrace factors and paradigms beyond the reductionistic framework of Western science or the RCT in the making of policies. This argument is made in the hope that the evidence-based paradigm will be reconceptualized to accept not only the evidence from systematic reviews of RCTs, but also that from studies which incorporate nonreducible sociocultural belief patterns, which themselves are crucial to the therapeutic process.
